# Vaccination frequency in people newly diagnosed with multiple sclerosis

**DOI:** 10.1177/13524585231199084

**Published:** 2023-10-13

**Authors:** Sonia Darvishi, Ewan Donnachie, Christiane Gasperi, Alexander Hapfelmeier, Bernhard Hemmer

**Affiliations:** Department of Neurology, Klinikum rechts der Isar, School of Medicine, Technical University of Munich, Munich, Germany; Bavarian Association of Statutory Health Insurance Physicians, Munich, Germany; Department of Neurology, Klinikum rechts der Isar, School of Medicine, Technical University of Munich, Munich, Germany; Institute of General Practice and Health Services Research, Technical University of Munich, Munich, Germany; Institute of AI and Informatics in Medicine, Technical University of Munich, Munich, Germany; Department of Neurology, Klinikum rechts der Isar, School of Medicine, Technical University of Munich, Munich, Germany; Munich Cluster for Systems Neurology (SyNergy), Munich, Germany

**Keywords:** Multiple sclerosis, vaccination, infection, diagnosis, Crohn’s disease, psoriasis

## Abstract

**Background:**

Infections are discussed as risk factor for multiple sclerosis (MS) development and relapses. This may lead to decreased vaccination frequency in newly diagnosed patients.

**Objective:**

The aim of this study was to evaluate the relation of MS diagnosis to subsequent vaccination frequency.

**Methods:**

Based on German ambulatory claims data from 2005 to 2019, regression models were used to assess the relation of MS diagnosis (*n* = 12,270) to vaccination. A cohort of patients with MS was compared to control cohorts with Crohn’s disease, psoriasis, and without these autoimmune diseases (total *n* = 198,126) in the 5 years after and before diagnosis.

**Results:**

Patients with MS were less likely to be vaccinated compared to persons without the autoimmune diseases 5 years after diagnosis (odds ratio = 0.91, *p* < 0.001). Exceptions were vaccinations against influenza (1.29, *p* < 0.001) and pneumococci (1.41, *p* < 0.001). Differences were strong but less pronounced after than before diagnosis (*p* < 0.001). The likelihood of vaccination was also lower compared to patients with Crohn’s disease or psoriasis.

**Conclusions:**

Patients with MS were not adequately vaccinated despite guideline recommendations. Increasing awareness about the importance of vaccination is warranted to reduce the risk of infection, in particular, in patients with MS receiving immunotherapies.

## Introduction

Multiple sclerosis (MS) is a chronic autoimmune inflammatory disease of the central nervous system (CNS) that affects 2.8 million people worldwide.^1^ Approximately 250,000 people in Germany are diagnosed with MS, with a prevalence rate of about 300 per 100,000 people.^2^ While the pathophysiology of MS is yet to be fully understood, several genetic and environmental risk factors for MS have been identified including Epstein–Barr Virus infection, low vitamin D levels, smoking, and obesity.^3–5^ A number of disease-modifying therapies (DMT) have been approved for the treatment of MS. These drugs affect the immune system and may increase the risk of infections including common viral and bacterial infections.^6–9^ Furthermore, relapses are associated with viral infections suggesting that infections can promote inflammatory disease activity in MS.^10,11^ All these findings strongly argue for specific precautions to avoid infectious diseases in people with MS.^12^

Over the last decades, the role of vaccination with respect to MS risk and the occurrence of relapses has been controversial^13–16^ although most controlled studies did not demonstrate an association of vaccination with relapses. Moreover, vaccination did not reveal to be a risk factor for developing MS.^17–22^ Several studies and guidelines argue that benefits of vaccination largely outweigh any potential risks^23^ and the benefit of vaccination for vaccine-preventable infections is evident.^24,25^ Despite the overwhelming evidence on the benefits, hesitant attitude toward vaccination has remained among patients and health care workers including physicians.^26^ How this affects vaccination behavior of patients newly diagnosed with MS is still uncertain.

The objective of this retrospective cohort study was to determine the vaccination frequency in the 5 years following a diagnosis of MS and to compare it with the frequency observed in patients with newly diagnosed Crohn’s disease and psoriasis, and matched controls with none of these autoimmune diseases (No AID). We also investigated whether vaccination frequencies in MS differ in the 5 years after compared to the 5 years before diagnosis.

## Methods

### Study design and population

We conducted a retrospective cohort study which compared the vaccination frequencies of a cohort of patients with MS to control cohorts with Crohn’s disease, psoriasis, and No AID, based on German ambulatory claims data from 2005 to 2019. Therefore, we defined a cohort of patients newly diagnosed with MS, which required at least two secure International Classification of Diseases (10th edition, ICD-10) diagnoses G35, a recorded visit with a neurologist, and no diagnosis of a clinically isolated syndrome (CIS, ICD-10 diagnosis G04). Two control cohorts consisted of patients diagnosed with Crohn’s disease or psoriasis, with the corresponding ICD-10 codes K50 and L40 recorded in at least two separate quarterly periods, respectively. A third control cohort of patients without any of the three autoimmune diseases was randomly selected (without replacement) from the Bavarian Association of Statutory Health Insurance Physicians (BASHIP) data. These patients were matched to the MS cohort in an approximate 3:1 ratio by birth year, sex, and district of residence. An index quarter to define the start of the observation period was set as the diagnosis date of the corresponding matching partner. We used three definitions of the cohorts to perform a main analysis, a sensitivity analysis, and a time-comparative analysis. In the main analysis, we included participants diagnosed before 2015 to be able to investigate follow-up periods of 5 years after diagnosis. For sensitivity analysis, we defined a reduced set excluding participants with potentially incomplete observation periods due to insurance starting or ending, for example, due to regional change of residence or death. For this, records had to be available for an insured person even before and including the first quarter and within or after the last quarter of the entire 5-year period after diagnosis. In addition, for the time-comparative analysis, another reduced set of participants diagnosed between 2010 and 2014 was defined to investigate the 5 years before and after diagnosis. To avoid assessing a period when the disease had potentially started, we excluded the quarter immediately prior to the diagnosis and used the 20 quarters preceding it. Age was restricted to 21–70 years to focus on adults and age groups with sufficient sample sizes for robust effect estimation. We excluded participants with a family doctor-centered care contract (German: hausarztzentrierte Versorgung, HZV). Approximately 21% of participants in all cohorts were enrolled in this contract at some point during the observation period, meaning that many family doctor claims—including vaccinations—were billed via a separate organization, and are therefore not covered by the BASHIP data. The participants with the HZV contract were less often female (57.3%) and they were predominantly older with an average age of 53.7 ± 17.8 years (cf. Table 1).

**Table 1.** Descriptive statistics of cohorts.

	MS	No AID	Crohn’s	Psoriasis
Main analysis (5 years after diagnosis), *n* (%)				
Size	12,270	35,920	19,523	142,683
Sex				
Female	8537 (69.5)	24,562 (68.3)	11,064 (56.6)	74,504 (52.2)
Age at first diagnosis (years)				
21–30	3059 (24.9)	8109 (22.6)	5448 (27.9)	22,413 (15.7)
31–40	3357 (27.3)	9597 (26.7)	4017 (20.5)	26,152 (18.3)
41–50	3338 (27.2)	9835 (27.4)	4483 (22.9)	33,498 (23.4)
51–60	1691 (13.7)	5394 (15)	3398 (17.4)	33,961 (23.8)
61–70	825 (6.7)	2985 (8.3)	2177 (11.1)	26,659 (18.6)
Time-comparative analysis (5 years before and 5 years after diagnosis), *n* (%)				
Size	8800	30,624	13,055	90,924
Sex				
Female	6097 (69.3)	20,879 (68.2)	7373 (56.5)	47,343 (52.1)
Age at first diagnosis (years)				
21–30	2318 (25.2)	7230 (22.6)	3869 (28.5)	15,061 (15.6)
31–40	2458 (26.9)	8410 (26.5)	2761 (20.2)	17,372 (18.1)
41–50	2476 (26.8)	8712 (27.1)	3068 (22.3)	22,487 (23.4)
51–60	1317 (14.4)	4888 (15.3)	2470 (18.2)	23,437 (24.4)
61–70	607 (6.6)	2647 (8.4)	1482 (10.8)	17,736 (18.4)

### Source of information and variables

BASHIP’s ambulatory claims data cover all the approximately 11 million members of Bavaria’s statutory health insurance, which accounts for about 85% of the population. It includes diagnoses coded according to the German version of ICD-10, sex (male/female), age, date of diagnosis, and information about vaccinations by service records (German: Gebührenordnungspositionen (GOP)) of quarterly reimbursement claims. Since vaccines are also administered in combination, we classified them into nine vaccine groups: (1) tick-borne encephalitis (TBE), (2) hepatitis A, (3) hepatitis B, (4) influenza virus, (5) meningococci, (6) MMR and VZV (MMRV), (7) pneumococci, (8) Clostridium tetani, Corynebacterium diphtheriae, poliovirus, Bordetella pertussis, and Hemophilus influenza type B (DPTPH), and (9) any vaccination. For members of the statutory health insurance, the listed vaccinations are free of charge since they are part of the set of vaccines recommended by the German Standing Committee on Vaccination (STIKO). Data were available for the years 2005–2019.

### Analysis

The distribution of data is presented by absolute and relative frequencies. Respective cumulative sums are used to explore trends in time.

In the main analysis, the association of a diagnosis of MS to any vaccination and each type of (combination) vaccination within the 5 years after diagnosis was investigated through unconditional logistic regression models. The binary outcome in each model was vaccination (yes = at least once /no = never), and the factorial covariates were the cohorts (MS/Crohn’s disease/psoriasis/No AID), and the main effects and the interaction effect of sex (male/female) and age (21–30, 31–40, 41–50, 51–60, 61–70 years). Results of the analysis are presented by odds ratios (ORs) with 95% confidence intervals (CIs). A sensitivity analysis of the main analysis was performed the same way using a reduced set of participants described above.

A time-comparative analysis was performed in the second reduced set of participants to explore whether observed differences in the vaccination frequencies could actually be related to the event of the diagnosis or existed even before. Therefore, we used generalized estimating equations (GEE) with an exchangeable covariance matrix^27^ to model the binary outcome vaccination (yes = at least once/no = never) in dependence of time (5 years before/5 years after diagnosis), the MS cohort and No AID cohort, and their interaction. Further covariates were age, sex, and their interaction. We used the “multicomp” package to perform linear hypothesis tests of differences in the obtained ORs of vaccination between the periods before and after diagnosis.^28^

Hypothesis testing was performed at exploratory two-sided 5% significance levels. Effect measures are presented with respective two-sided 95% CIs. All analyses were conducted using R 4.0.2 (The R Foundation for Statistical Computing, Vienna, Austria).

## Results

**Figure 1.**
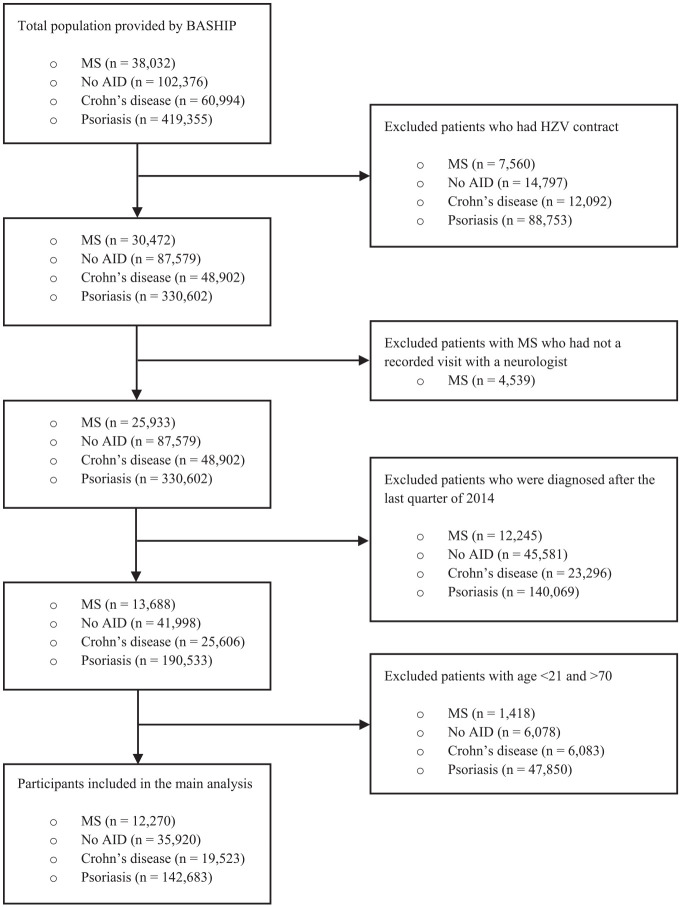
Flowchart of selection of the study populations.

The data provided by BASHIP contained 38,032 participants newly diagnosed with MS, 60,994 participants newly diagnosed with Crohn’s disease, 419,355 participants newly diagnosed with psoriasis, and 102,376 participants with No AID between 2008 and 2019. These sample sizes decreased to 12,270 patients with MS, 19,523 patients with Crohn’s disease, 142,683 patients with psoriasis, and 35,920 participants with No AID when applying the previously mentioned definitions of the study cohorts to ensure that only patients were included with secured diagnosis and diagnosed between 2008 and 2014 and to ensure a follow-up of 5 years after diagnosis (see “Methods” section and Figure 1).

Descriptive statistics of the cohorts are detailed in Table 1 (respective information about the reduced sets of participants used in the sensitivity analysis is given in Supplementary Table S1).

**Figure 2.**
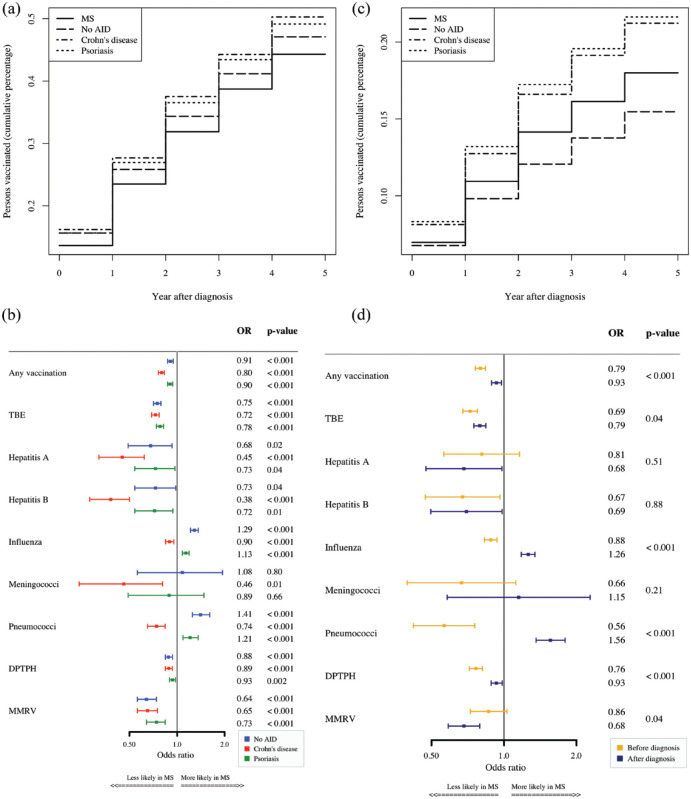
(a) Cumulative sum of percentage of vaccinated persons by year. (b) ORs of vaccination for patients with MS versus control cohorts. (c) Cumulative sum of percentage of persons vaccinated against influenza by year. (d) Time-comparative analysis of ORs of vaccinations 5 years before and after diagnosis for MS cohort versus No AID.

In general, patients with MS had a low vaccination rate with only 44.8% of all patients with MS having received at least one vaccination in the 5 years after first diagnosis. Furthermore, they were vaccinated less frequently compared to patients with Crohn’s disease (50.7%), patients with psoriasis (49.5%), and patients with No AID (47.5%) in the 5 years after diagnosis. A year-by-year analysis for any vaccination revealed that these differences emerge from minor differences that accumulate over the 5-year period (see Figure 2(a), Table 2).

**Table 2.** Number of vaccinated persons in cohorts in the 5 years after diagnosis.

	MS, *n* (%)	No AID, *n* (%)	Crohn’s disease, *n* (%)	Psoriasis, *n* (%)
Size	12,270	35,920	19,523	142,683
Any vaccination	5497 (44.8)	17,086 (47.5)	9908 (50.7)	70,724 (49.5)
Tick-borne encephalitis	2442 (19.9)	8990 (25)	4947 (25.3)	35,462 (24.8)
Hepatitis A	47 (0.3)	196 (0.5)	165 (0.8)	661 (0.4)
Hepatitis B	56 (0.4)	219 (0.6)	232 (1.2)	834 (0.6)
Influenza	2208 (17.9)	5557 (15.5)	4143 (21.2)	30,865 (21.6)
Meningococci	14 (0.1)	38 (0.1)	49 (0.2)	186 (0.1)
Pneumococci	362 (2.9)	833 (2.3)	932 (4.7)	5882 (4.1)
Diphtheria, pertussis, tetanus, poliomyelitis, Hemophilus influenza type B	3165 (25.8)	10,131 (28.2)	5458 (27.9)	38,228 (26.8)
Measles, mumps, rubella, varicella	264 (2.1)	1119 (3.1)	559 (2.8)	2667 (1.8)

The adjusted ORs for any vaccination were 0.80 (95% CI 0.76–0.83), 0.90 (95% CI 0.87–0.94), and 0.91 (95% CI 0.87–0.94) with respect to the cohort with Crohn’s disease, to the cohort with psoriasis, and to the cohort of No AID, respectively (Figure 2(b)).

Overall, the odds of vaccinations in the 5 years after diagnosis in the cohort of patients with MS were significantly lower than in other control cohorts for vaccinations against TBE, Hepatitis A, Hepatitis B, DPTPH, MMRV, and any vaccination. Two results deviating from this showed that the odds of being vaccinated against influenza and pneumococci within the 5 years after diagnosis were higher among patients with MS than in patients with No AID and patients with psoriasis. Results obtained from the sensitivity analyses were similar (see Supplementary Figure S1). A year-by-year analysis revealed that this difference remained rather stable over the 5-year period (Figure 2(c)).

Next, we compared vaccination frequencies in MS in the 5 years before and after diagnosis (Table 3). The ORs of vaccination frequencies in patients with MS compared to the No AID cohort are different in the periods before and after diagnosis (*p* < 0.001). The ORs for any vaccination, TBE, influenza, pneumococci, DPTPH, and MMRV increased after diagnosis in the comparison to the No AID cohort (*p* < 0.001), but except for vaccinations against influenza and pneumococci, the ORs were still below 1.0 showing a lower vaccination frequency in patients with MS as compared to the No AID cohort (see Figure 2(d)).

**Table 3.** Frequency of vaccinated patients with MS before and after diagnosis.

Sex	Female					Male				
Age at first diagnosis (years)	21–30	31–40	41–50	51–60	61–70	21–30	31–40	41–50	51–60	61–70
Size	1594	1622	1670	840	371	621	749	693	425	215
Before diagnosis, *n* (%)										
Any vaccination	603 (37.8)	623 (38.4)	654 (39.1)	361 (42.9)	199 (53.6)	179 (28.8)	234 (31.2)	242 (34.9)	191 (44.9)	115 (53.5)
TBE	202 (12.7)	220 (13.5)	209 (12.5)	124 (14.7)	66 (17.8)	67 (10.8)	69 (9.2)	68 (9.8)	59 (13.9)	42 (19.5)
Hepatitis A	10 (0.6)	9 (0.5)	7 (0.4)	2 (0.2)	0	3 (0.4)	5 (0.6)	0	0	0
Hepatitis B	10 (0.6)	8 (0.5)	6 (0.3)	4 (0.5)	0	4 (0.6)	3 (0.4)	1 (0.1)	0	0
Influenza	274 (17.2)	294 (18.1)	363 (21.7)	215 (25.6)	157 (42.3)	70 (11.3)	113 (15.1)	128 (18.5)	113 (26.6)	95 (44.2)
Meningococci	6 (0.4)	3 (0.2)	2 (0.1)	2 (0.2)	0	0	1 (0.1)	3 (0.4)	0	0
Pneumococci	4 (0.2)	1 (0.1)	9 (0.5)	6 (0.7)	13 (3.5)	2 (0.3)	3 (0.4)	2 (0.3)	5 (1.1)	7 (3.2)
Diphtheria, pertussis, tetanus, poliomyelitis, Hemophilus influenza type B	272 (17.1)	284 (17.5)	278 (16.6)	145 (17.3)	61 (16.4)	90 (14.5)	124 (16.5)	108 (15.6)	85 (20)	35 (16.3)
Measles, mumps, rubella, varicella	44 (2.7)	45 (2.7)	17 (1)	8 (0.9)	11 (2.9)	10 (1.6)	15 (2)	5 (0.7)	3 (0.7)	4 (0.2)
After diagnosis, *n* (%)										
Any vaccination	730 (45.8)	706 (43.5)	748 (44.8)	430 (51.2)	226 (60.9)	248 (39.9)	301 (40.2)	290 (41.8)	218 (51.3)	139 (64.6)
TBE	330 (20.7)	360 (22.2)	338 (20.2)	187 (22.2)	104 (28)	113 (18.2)	126 (16.8)	138 (19.9)	106 (24.9)	72 (33.5)
Hepatitis A	13 (0.8)	5 (0.3)	7 (0.4)	1 (0.1)	0	3 (0.5)	4 (0.5)	2 (0.3)	0	0
Hepatitis B	9 (0.5)	7 (0.4)	7 (0.4)	3 (0.3)	0	7 (1.1)	4 (0.5)	3 (0.4)	0	0
Influenza	214 (13.4)	213 (13.1)	290 (17.3)	213 (25.3)	157 (42.3)	61(9.8)	90 (12)	112 (16.1)	132 (31.1)	100 (46.5)
Meningococci	1 (0.1)	1 (0.1)	3 (0.2)	1 (0.1)	1 (0.3)	1 (0.1)	0	0	2 (0.5)	1 (0.5)
Pneumococci	25 (1.5)	40 (2.5)	32 (1.9)	49 (5.8)	50 (13.5)	11 (1.8)	10 (1.3)	19 (2.7)	33 (7.8)	28 (13)
Diphtheria, pertussis, tetanus, poliomyelitis, Hemophilus influenza type B	487 (30.5)	443 (27.3)	450 (26.9)	233 (27.7)	103 (27.7)	178 (28.6)	189 (25.2)	189 (27.3)	110 (25.9)	62 (28.8)
Measles, mumps, rubella, varicella	70 (4.4)	69 (4.2)	15 (0.9)	2 (0.2)	1 (0.3)	17 (2.7)	25 (3.3)	10 (1.4)	2 (0.5)	1 (0.5)

## Discussion

In our cohort study, we investigated the relation of MS diagnosis to the frequency of vaccinations in the 5 years after diagnosis in comparison to three control cohorts of patients with Crohn’s disease, with psoriasis, and No AID. We also compared the relations studied with those in the 5 years before diagnosis to determine whether they were time-consistent and thus not dependent on diagnosis. We used a large, high-quality database covering 85% of the Bavarian population. We analyzed the frequencies of 15 vaccines and grouped them into nine sets, which covered all vaccinations recommended in Germany for patients with MS. Concerning the guidelines recommendation, our results indicate vaccination frequencies in patients with MS were generally lower than expected in the 5 years after diagnosis. Furthermore, there was a lower likelihood of vaccination in patients with MS in the 5 years after diagnosis compared to all three control cohorts. However, 44.8% of patients with MS, 50.7% of patients with Crohn’s disease, 49.5% of patients with psoriasis, and 47.5% of patients with No AID were vaccinated in the 5 years after diagnosis. When analyzing single vaccinations, we could observe that compared to No AID, patients with MS had lower vaccination frequencies for DPTPH and MMRV, hepatitis A and B, and TBE. We only observed higher frequencies of vaccination for influenza and pneumococcal while almost no difference was seen for meningococci compared to the No AID group. Vaccination frequencies in the psoriasis control cohort were very similar to the No AID group, and while the vaccination frequencies were overall higher in the Crohn’s cohort, we still observed similar results when comparing patients with MS to these two control cohorts. The difference in vaccination frequencies between MS and the control cohorts increased over time and reached the highest value 5 years after diagnosis.

International and national guidelines recommend that patients with MS should follow local vaccine standards to prevent infections.^24,29,30^ These recommendations take into account that many patients with MS receive DMTs which may go along with an increased rate of infection, which can be effectively prevented by vaccination.

To our knowledge, this is the first study that investigated vaccination frequency in patients diagnosed with MS in a systematic manner covering a large proportion of the population and several well-selected control cohorts. Previous studies focused on seasonal/lifetime vaccination rates of selected vaccines in selected cohorts and were often based on questionnaires rather than physician-based documentation of vaccine procedures.^31,32^ The lack of literature with comparable results makes it difficult to compare our results with other publications. A recent study in Germany published seasonal and lifetime vaccination rates for influenza, pneumococcal, TBE, MMRV, and DPTPH vaccines. Roughly converting these results to the length of the present study period, the results are consistent with our findings.^33^

Several factors may account for the lower frequency of vaccinations in patients with MS in the 5 years after diagnosis. The idea that viral or bacterial infections play an important role in causing and perpetuating MS has been promoted by several studies.^34^ Moreover, there were public discussions on the role of hepatitis, measles, and TBE vaccines for MS or other autoimmune diseases of the CNS.^35,36^ However, well-designed retrospective studies and prospective trials have not found evidence that vaccination is associated with MS risk, relapses, or disease progression. Indeed practice guidelines from North America and Europe concluded that there are no sufficient studies to support or refute the claim that MS development is associated with vaccinations against diphtheria, hepatitis B, influenza, measles, mumps, measles–mumps–rubella, VZV, poliomyelitis, and rubella.^24^ The guidelines recommend to follow local vaccination recommendations but strongly recommend influenza vaccination.

Nevertheless, half of unvaccinated patients are concerned about whether the vaccine would worsen their MS or general adverse effects.^31^ A recent study from Germany showed that about 70% of patients with MS stated that they did not receive a consultation regarding the vaccination over the past years.^37^

As reported previously by our group, vaccination frequencies are already lower during the prodromal phase before MS diagnosis.^17^ Several studies have demonstrated that unspecific symptoms, behavioral changes, and neuropsychiatric symptoms can occur years before MS diagnosis. This may also impact on vaccination behavior of persons during the prodromal MS phase. Interestingly, the MS diagnosis may have a specific impact on some vaccinations.

Likewise, higher frequency of influenza and pneumococcus vaccinations is observed among patients with MS in the 5 years after diagnosis compared to No AID and the vaccination frequencies increase when comparing time frames before and after MS diagnosis. It may not only be the result of specific guideline recommendations^24,29,30^ but also the knowledge of the well-known association of respiratory infections and relapses^13,17,21,38^. The higher frequency of influenza vaccination indicates that specific recommendations may have indeed reached clinical practice. However, numbers are still low and do not demonstrate that a significant proportion of patients receive a yearly influenza vaccination. By contrast, MMR vaccine use decreases when comparing time frames before and after diagnosis. This may be explained by the nature of the vaccine. MMR vaccine is a live-attenuated vaccine and should not be administered in patients receiving immunosuppressive drugs. Since many patients are treated with DMTs or consider DMTs, they might refer from receiving live-attenuated vaccines.

Overall, our study supports the assumption that patients newly diagnosed with MS have low vaccination frequencies despite scientific evidence strongly supporting vaccination in patients with MS. While the differences in vaccination rates between patients with MS and the No AID cohort became smaller after diagnosis (we observed lower vaccination rates for the MS cohort also in the 5 years before diagnosis), the vaccination rates of patients with MS were overall still lower as compared to controls and thereby do not meet the recommendations and remain below the minimum requirements. This is highly important because most patients receive DMTs. In particular, the more recently introduced DMTs are highly efficient but may go along with an increased frequency of infections some of them even life-threatening.^8^ Because some of the DMTs decrease vaccine responses (e.g. S1P modulators and CD20 antibodies), it is recommended to refresh or complete the vaccination status before initiating these DMTs. Therefore, rather higher than lower vaccination frequencies would be expected in patients newly diagnosed with MS compared to control cohorts.

Our study emphases the importance of increasing efforts by physicians to discuss with patients the evidence related to vaccination in MS and strongly recommend vaccination unless there is a specific individual contraindication. This is particularly true for patients who will be treated with more effective DMTs.

This study has limitations due to the data source itself. It is impossible to eliminate entry errors and incorrect coding, even in databases that adhere to high standards, such as the BASHIP database.

Furthermore, certain patients had to be excluded from the analysis as they were part of the HZV program, which allowed treating physicians to be exempted from coding diagnoses and procedures. Consequently, there is a possibility that the frequencies of certain outcomes may be underestimated, particularly with regard to older participants who were supposed to be vaccinated but were excluded from the analysis. In addition, there is a potential that certain patients with MS were already present in the 5-year period preceding their diagnosis. We tried to mitigate their influence by excluding data from the last quarter before diagnosis and cases diagnosed as CIS.

## Conclusion

Vaccination frequencies are low in patients newly diagnosed with MS and also lower than the frequencies observed for control cohorts with or without other autoimmune diseases. Additional efforts are warranted to inform patients and caregivers about the importance of vaccination in MS to increase vaccination frequencies impacting on the risk of relapse and preventable infections in patients with MS in particular those on DMTs.

## Supplemental Material

sj-docx-1-msj-10.1177_13524585231199084 – Supplemental material for Vaccination frequency in people newly diagnosed with multiple sclerosis Click here for additional data file. Supplemental material, sj-docx-1-msj-10.1177_13524585231199084 for Vaccination frequency in people newly diagnosed with multiple sclerosis by Sonia Darvishi, Ewan Donnachie, Christiane Gasperi, Alexander Hapfelmeier and Bernhard Hemmer in Multiple Sclerosis Journal

sj-tiff-2-msj-10.1177_13524585231199084 – Supplemental material for Vaccination frequency in people newly diagnosed with multiple sclerosis Click here for additional data file. Supplemental material, sj-tiff-2-msj-10.1177_13524585231199084 for Vaccination frequency in people newly diagnosed with multiple sclerosis by Sonia Darvishi, Ewan Donnachie, Christiane Gasperi, Alexander Hapfelmeier and Bernhard Hemmer in Multiple Sclerosis Journal
