# Supplementary material for: Vaccination frequency in people newly diagnosed with multiple sclerosis
Source: Mult Scler. 2023 Oct 13;29(14):1831–40. doi: 10.1177/13524585231199084 (PMC10687801; doi:10.1177/13524585231199084)

**Table S1** Descriptive statistics of cohorts used in the sensitivity analysis

|  |  | **MS, n (%)** | **No AID, n (%)** | **Crohn’s, n (%)** | **Psoriasis, n (%)** |
| --- | --- | --- | --- | --- | --- |
| **Sensitivity analysis (5 years after diagnosis)** | | |  |  |  |
|  | Size | 11596 | 32982 | 18026 | 131394 |
|  | Sex |  |  |  |  |
|  | Female | 8093 (69.8) | 22758 (69) | 10243 (56.8) | 69510 (52.9) |
|  | Age at first diagnosis |  |  |  |  |
|  | 21-30 | 2867 (24.7) | 7131 (21.6) | 4939 (27.4) | 19912 (15.1) |
|  | 31-40 | 3195 (27.5) | 8824 (26.7) | 3761 (20.8) | 24268 (18.5) |
|  | 41-50 | 3199 (27.6) | 9323 (28.2) | 4251 (23.6) | 31727 (24.1) |
|  | 51-60 | 1599 (13.8) | 5062 (15.3) | 3160 (17.5) | 31774 (24.2) |
|  | 61-70 | 736 (6.3) | 2642 (8) | 1915 (10.6) | 23713 (18.1) |

**Figure S1** Odds ratios (ORs) of vaccination for patients with MS vs. control cohorts in the sensitivity analysis


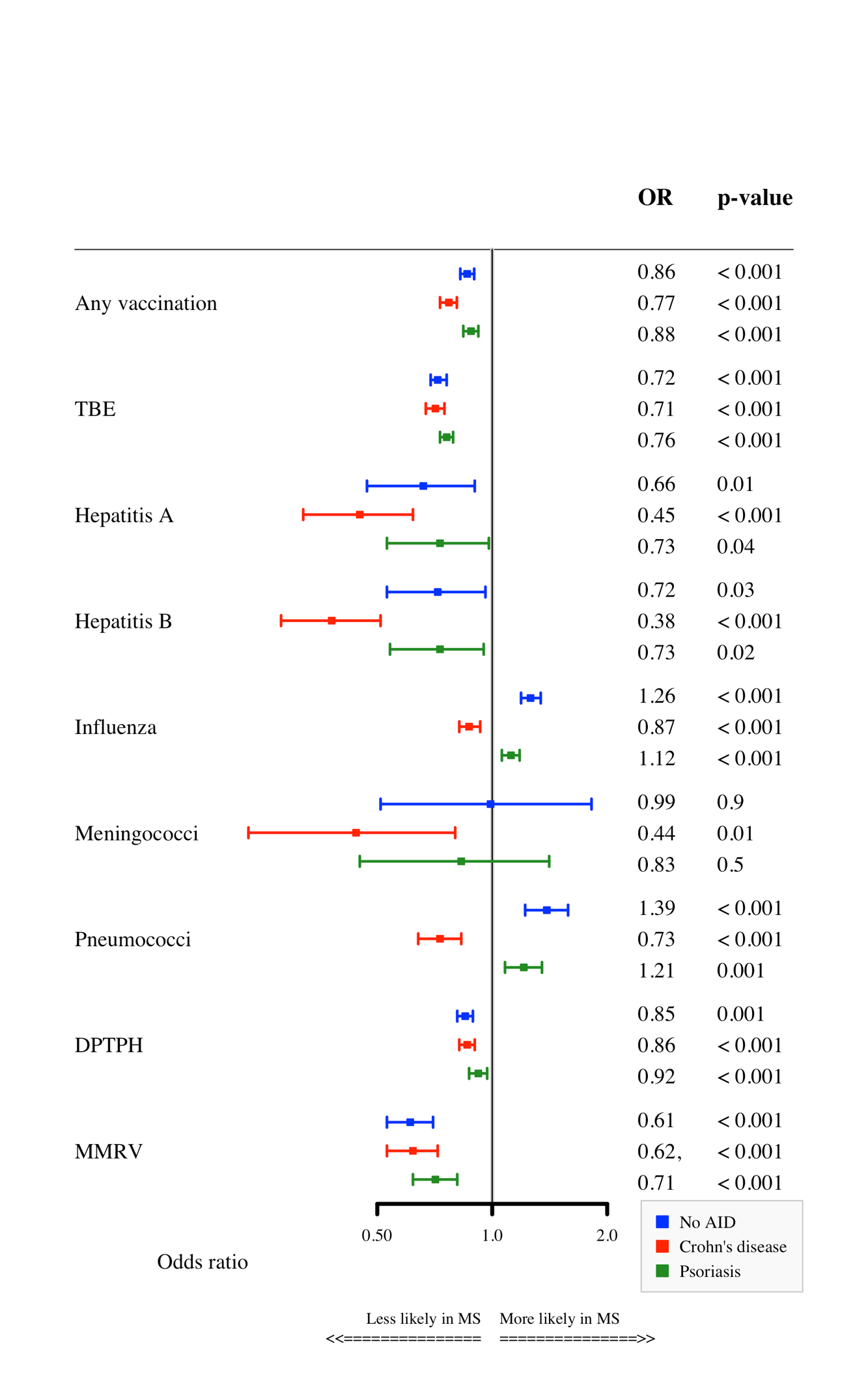

Supplement: sj-docx-1-msj-10.1177_13524585231199084 – Supplemental material for Vaccination frequency in people newly diagnosed with multiple sclerosis [file sj-docx-1-msj-10.1177_13524585231199084.docx]
