# Supplementary figures and images for: Vaccination frequency in people newly diagnosed with multiple sclerosis
Source: Mult Scler. 2023 Oct 13;29(14):1831–40. doi: 10.1177/13524585231199084 (PMC10687801; doi:10.1177/13524585231199084)

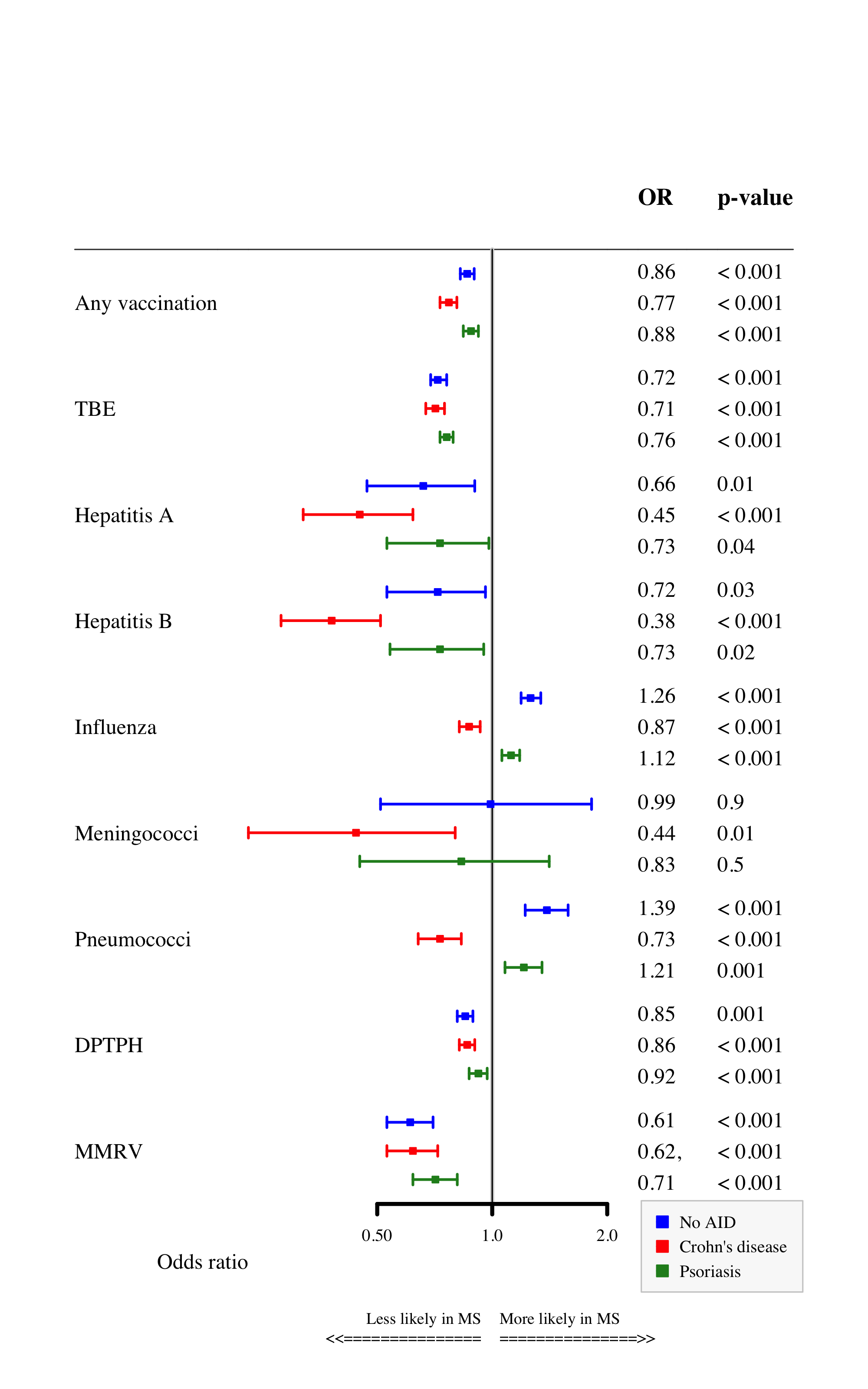

Supplement: sj-tiff-2-msj-10.1177_13524585231199084 – Supplemental material for Vaccination frequency in people newly diagnosed with multiple sclerosis [file sj-tiff-2-msj-10.1177_13524585231199084.tiff]
